# Estimation of tuna population by the improved analytical pipeline of unique molecular identifier-assisted HaCeD-Seq (haplotype count from eDNA)

**DOI:** 10.1038/s41598-021-86190-6

**Published:** 2021-04-12

**Authors:** Kazutoshi Yoshitake, Atushi Fujiwara, Aiko Matsuura, Masashi Sekino, Motoshige Yasuike, Yoji Nakamura, Reiichiro Nakamichi, Masaaki Kodama, Yumiko Takahama, Akinori Takasuka, Shuichi Asakawa, Kazuomi Nishikiori, Takanori Kobayashi, Shugo Watabe

**Affiliations:** 1 Department of Aquatic Bioscience, Graduate School of Agricultural and Life Sciences, The University of Tokyo, 1-1-1 Yayoi, Bunkyo-ku, Tokyo, 113-8657 Japan; 2 Fisheries Technology Institute, Japan Fisheries Research and Education Agency, 422-1 Nakatsuhamaura, Minami-ise, Mie, 516-0193 Japan; 3 Fisheries Resources Institute, Japan Fisheries Research and Education Agency, 2-12-4 Fuku-ura, Kanazawa, Yokohama, Kanagawa 236-8648 Japan; 4 Tokyo Sea Life Park, 6-2-3 Rinkai-cho, Edogawa-ku, Tokyo, 134-8587 Japan; 5 Japan Fisheries Research and Education Agency, 1-1-25 Shinurashima-cho, Kanagawa-ku, Yokohama, Kanagawa 221-8529 Japan; 6 School of Marine Biosciences, Kitasato University, 1-15-1 Kitasato, Minami-ku, Sagamihara, Kanagawa 252-0373 Japan

**Keywords:** Ecology, Molecular biology, Zoology, Ecology

## Abstract

Many studies have investigated the ability to identify species from environmental DNA (eDNA). However, even when individual species are identified, the accurate estimation of their abundances by traditional eDNA analyses has been still difficult. We previously developed a novel analytical method called HaCeD-Seq **(H**aplotype **C**ount from **eD**NA), which focuses on the mitochondrial D-loop sequence. The D-loop is a rapidly evolving sequence and has been used to estimate the abundance of eel species in breeding water. In the current study, we have further improved this method by applying unique molecular identifier (UMI) tags, which eliminate the PCR and sequencing errors and extend the detection range by an order of magnitude. Based on this improved HaCeD-Seq pipeline, we computed the abundance of Pacific bluefin tuna (*Thunnus orientalis*) in aquarium tanks at the Tokyo Sea Life Park (Kasai, Tokyo, Japan). This tuna species is commercially important but is at high risk of resource depletion. With the developed UMI tag method, 90 out of 96 haplotypes (94%) were successfully detected from Pacific bluefin tuna eDNA. By contrast, only 29 out of 96 haplotypes (30%) were detected when UMI tags were not used. Our findings indicate the potential for conducting non-invasive fish stock surveys by sampling eDNA.

## Introduction

The accessibility of new molecular techniques, such as high-throughput sequencing, has enabled the assessment of biodiversity wherein samples are noninvasively acquired from various environments, including aquatic environments. These samples are cells released into the environment^1–4^ and there are many studies for estimation of biomass by using DNA extracted from these cells, called environmental DNA (eDNA)^5–7^. However, the method using eDNA reported previously had some limitations. One of the problems when studying eDNA is that different quantities of cells are released into aqueous environments from different individuals even in the same species in the same environment due to variations in size and age-class distributions^8^ as well as alteration in environmental factors such as water temperature and flow^9^. We have recently developed a novel method for the reliable and simple estimation of fish populations abundance from eDNA using the haplotype count^10^. This method, called HaCeD (**Ha**plotype **C**ount from **eD**NA)-Seq, was successfully adopted to noninvasively estimate the abundances of various eel species inhabiting the same experimental tank as well as the aquaculture pond at the same time^10^.

In HaCeD-Seq, the accuracy of base identification in the sequencing data is very important. Even a single base difference will be counted as a different haplotype in this method; thus, only reads with a base identification accuracy of 99.9% or higher were used in the previous report on eels^10^. However, even with 99.9% base identification accuracy, the probability of error occurrence at least once in every 300 bases (= the average length of a read) is (1–0.999^300^) = 26%. Therefore, even 99.9% accuracy is not high enough for this method. In addition, when samples contain several individuals, there are variations in the yield of eDNA derived from each individual as described above. It is generally difficult to discriminate between true minor haplotypes and false haplotypes derived from sequencing errors of the major haplotypes. In our previous report, due to sequencing errors, eels with less than 3% of those having the major haplotypes were indistinguishable from those with false haplotypes^10^.

Safe-SeqS is a sequencing technique that uses unique molecular identifiers (UMIs) to remove sequencing errors^11^. This technique assigns a random UMI tag to each DNA fragment, amplifies the fragment by PCR, sequences the amplified product and gathers the reads with the same UMI tag during data analysis, thus removing sequencing errors. Numerous studies have used UMI tags to remove PCR amplification bias, mainly in single-cell analysis^12,13^. Using UMI tags to remove PCR errors and sequencing errors has been adopted to analytical tools such as pRESTO^14^, MAGERI^15^ and AmpUMI^16^, which facilitate the assembly of high-quality reads from sequenced data. Thus, it is interesting to explore whether UMI tags enable HaCeD-Seq to correctly detect the abundance of individuals, even when the abundance is low.

HaCeD-Seq was established to determine population sizes, and it is applicable to organisms with individual specific D-loop sequences, namely, those with high haplotype diversity. The haplotype diversities of the D-loop of tuna are reportedly as high as 0.9980 for longtail tuna^17^, 0.9987–1.000 for yellowfin tuna^17,18^, and 0.996 for Pacific bluefin tuna^18^. The Tokyo Sea Life Park in Japan planned to add naturally harvested Pacific bluefin tuna juveniles to this aquarium in 2016. This was a good opportunity to validate the improved UMI-assisted HaCeD-Seq method, because fin tissue samples could be taken for the determination of D-loop haplotype before they were transferred into the aquarium.

Tuna are top predators with unique features in terms of swimming ability^19^. These fish continuously swim at a high speed to extract oxygen with their gills and to chase prey. Tuna has long been an international market commodity for canning^20^. The fast muscle of tuna is also consumed raw in fish dishes (sashimi); formerly, this consumption was almost exclusively in Japan but is now common throughout the world. The consumption of tuna has greatly increased owing to the desirable taste of sashimi. This fish also has high contents of unsaturated n-3 fatty acids, such as eicosapentaenoic acid (EPA, 20:5 n-3) and docosahexaenoic acid (DHA, 22:6 n-3), which are thought to be beneficial for human health^21,22^.

Due to the increasing demand from consumption, the tuna stocks are considered to be at risk of overexploitation, although it previously had been thought that tuna harvesting would never cause their populations to become threatened^20,23^. In response to the risk of over fishing, the aquaculture programme of the Pacific bluefin tuna started in 1970 in Japan, resulting in predominantly farm-raised fish by 2002^24,25^. Despite this innovative achievement, most tuna aquaculture business still depends on wild stocks of juveniles because the survival rates of larval and juvenile tuna from artificial reproduction is not high^26^. Therefore, it is urgent to assess natural tuna stocks correctly and easily.

The objective of this study was to improve our HaCeD-Seq method by combining UMI assembles and to examine the accuracy of this improved method to estimate the abundance of Pacific bluefin tuna that were reared in a large aquarium tank in the Tokyo Sea Life Park.

## Results

### UMI tagging to improve base accuracy

**Figure 1:**
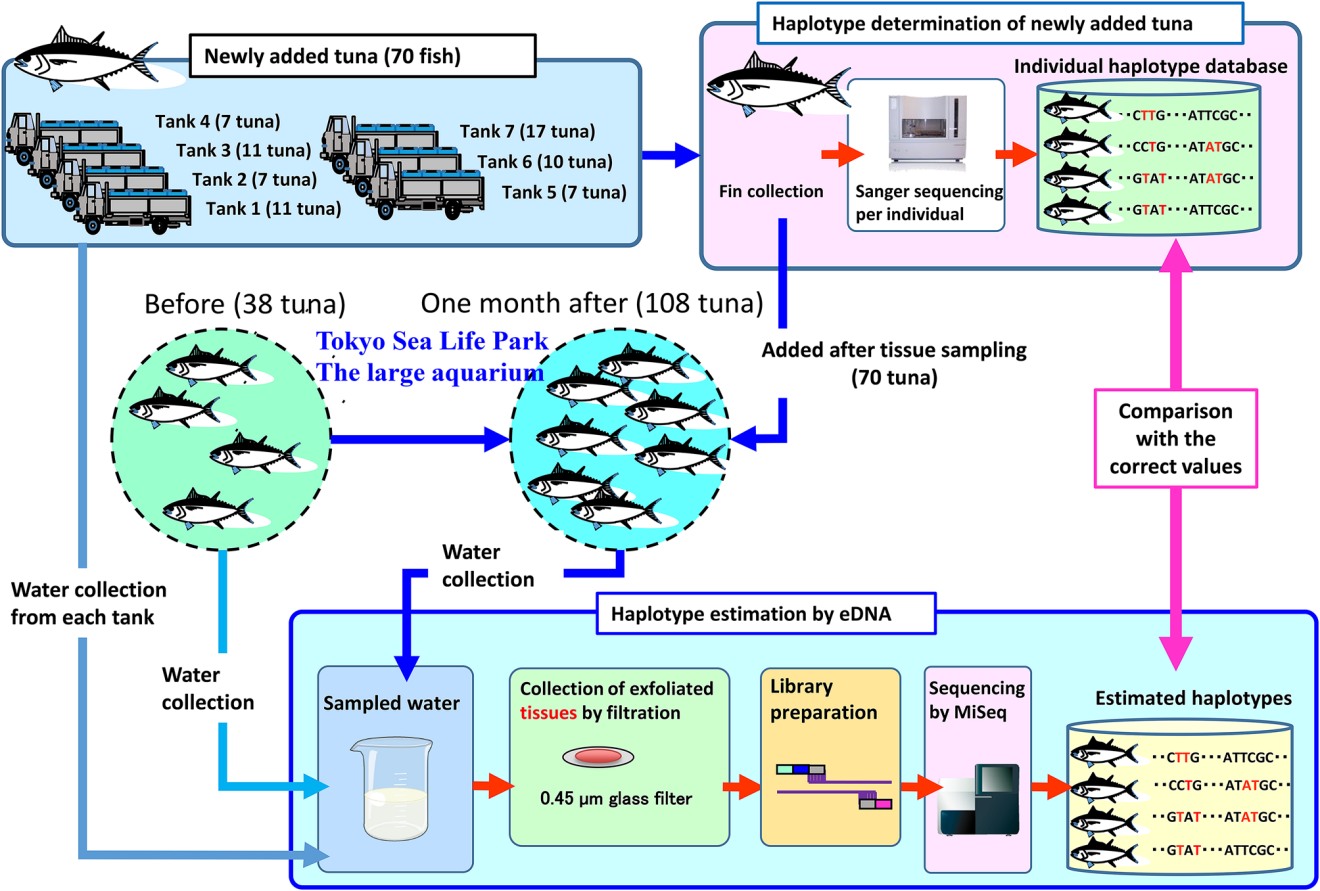
Experimental design of the study. Seventy Pacific bluefin tuna caught in the Pacific Ocean off Shikoku Island, Japan, were transported to the Tokyo Sea Life Park at Kasai, Tokyo. eDNA was extracted from water taken from the live fish vehicle tanks and subjected to HaCeD-Seq. The D-loop sequences of the 70 fish were determined by Sanger sequencing using DNA extracted from dorsal fin tissue samples that had been dissected with small forceps before the fish were added to the aquarium. One month after the 70 tuna were added to the large aquarium tank, water was sampled from the tank and the number of haplotypes was determined by HaCeD-Seq. The images of sequencers are from TogoTV (©2016 DBCLS TogoTV/CC-BY-4.0).

eDNA was extracted from water samples taken from a total of 70 Pacific bluefin tuna transported to the Tokyo Sea Life Park with 7 live fish transport vehicles (Fig. 1). This was only one opportunity to obtain samples in our experiments.

**Figure 2:**
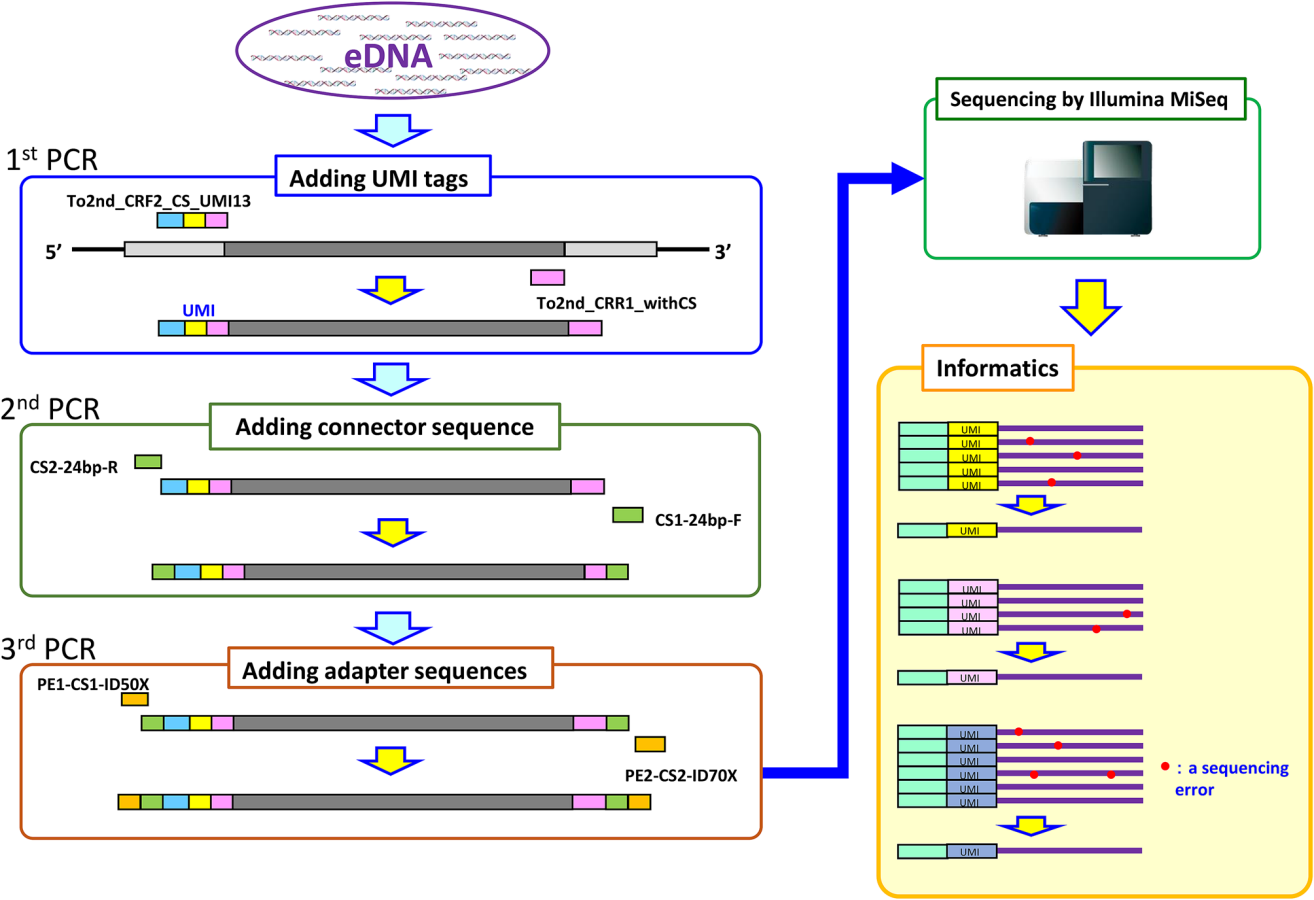
Principle of eliminating PCR errors and sequencing errors using unique molecular identifier (UMI) tags. Assign one random UMI tag per molecule in the first PCR; amplify by including the UMI tag in the second PCR; sequence with MiSeq; collect reads with the same UMI tag; perform error correction by removing different bases. The image of a sequencer is from TogoTV (©2016 DBCLS TogoTV/CC-BY-4.0).

Using the extracted eDNA as a template, PCR was performed with and without UMI-tagged primers, and the amplified products were sequenced by MiSeq at 300 bp × 2 paired-end sequencing (Fig. 2). The number of reads obtained is summarized in Tables 1 and 2. For sequencing data without the UMI tags, only high precision reads with all bases above Q30 (equal to 99.9% accuracy) were extracted, with an average of 32% of the reads remaining (Table 1). The average quality of bases in the obtained filtered reads was 37.9 (99.98% accuracy). The UMI-tagged reads were error-corrected by MAGERI, resulting in an average of 6% of the reads remaining (Table 2). The average quality of the bases was 39.8 (99.99% accuracy), but the real base accuracy was much higher since the upper limit of base quality output by MAGERI is up to 40.

**Table 1:** Statistics on sequence data without the UMI tags. The number of reads and the number and percentage of reads with all bases above Q30 are shown.

Tank	Individuals	Paired-end reads	> Q30 reads	Remaining reads (%)	Bases of > Q30 reads	Average base quality of > Q30 reads
**Transport vehicles**						
No. 1	11	3,84,457	1,20,193	31.3	4,38,66,841	37.94
No. 2	7	2,23,461	71,883	32.2	2,62,32,822	37.94
No. 3	11	4,47,696	1,50,634	33.6	5,49,41,606	37.95
No. 4	7	3,51,886	1,09,673	31.2	4,00,28,810	37.94
No. 5	7	2,53,536	83,057	32.8	3,03,15,443	37.95
No. 6	10	4,06,575	1,27,593	31.4	4,65,70,918	37.94
No. 7	17	5,48,745	1,76,627	32.2	6,44,67,585	37.94
**Aquarium**						
Before addition	38	14,47,500	4,79,641	33.1	17,50,66,441	37.95
After addition	108	42,96,541	13,87,557	32.3	50,64,41,781	37.95

**Table 2:** Statistics on sequence data with the UMI tags. The number of reads and the number and percentage of reads assembled by MAGERI are shown.

Tank	Paired-end reads	Assembled reads by MAGERI	Remaining reads (%)	Bases of assembled reads by MAGERI	Average base quality of assembled reads by MAGERI
**Transport vehicles**					
No. 1	3,19,040	17,048	5.3	69,34,013	39.86
No. 2	1,61,459	3132	1.9	12,74,727	39.85
No. 3	3,84,519	37,523	9.8	1,52,23,580	39.87
No. 4	1,85,995	234	0.1	95,584	39.50
No. 5	2,25,437	8074	3.6	32,85,460	39.86
No. 6	1,60,608	5416	3.4	22,04,473	39.86
No. 7	3,29,380	30,637	9.3	1,24,60,852	39.87
**Aquarium**					
Before addition	10,48,043	87,244	8.3	3,54,64,075	39.85
After addition	26,30,623	2,49,697	9.5	10,15,28,051	39.87

### Estimation of tuna abundances in water tanks of live fish transport vehicles

**Figure 3:**
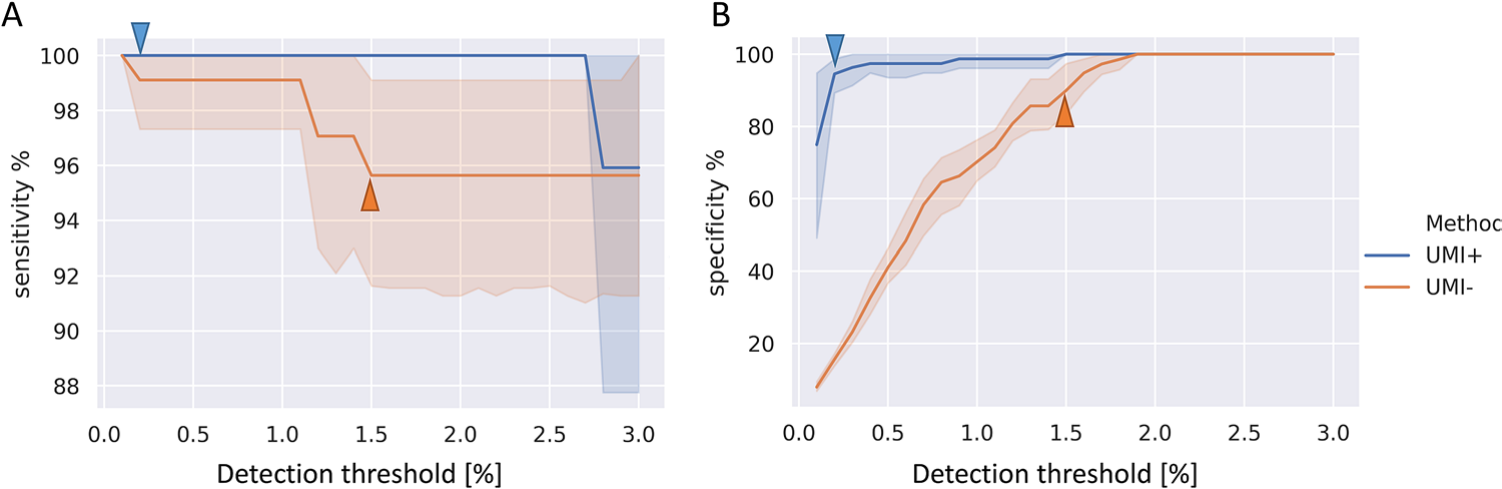
Differences in sensitivity and specificity with and without the UMI tags. (**A**) The relationship between the detection threshold and sensitivity changed depending on whether the UMI tags were used and depending on the detection threshold for the cluster size. Here, the sensitivity is the percentage of correct haplotypes among the total haplotypes observed. (**B**) The relationship between the detection threshold and specificity. Here, the specificity is the percentage of correct haplotypes compared to all the detected haplotypes. The horizontal axis shows the detection threshold, which is the size of the cluster when the most abundant haplotype is set to 100%. A threshold that was as low as possible without decreasing specificity was selected. The threshold finally adopted by HaCeD-Seq with the UMI tags (0.2%) is indicated by a blue triangle; the threshold adopted without the UMI tags (1.5%) is indicated by an orange triangle.

Each of the seven tanks in the transport vehicles contained 7–17 tuna specimens (Table 1). When the D-loop sequences of all 70 individuals were determined by Sanger sequencing, duplicated sequences were found for two sets, whereas one sequence was found for three individuals. Thus, in total, 64 haplotypes were found for the 70 fish in the tanks (Table 3, Supplementary Information Table S1). The sequences of 64 haplotypes determined by Sanger sequencing were identical to those determined by NGS with and without the UMI tags. We also performed HaCeD-Seq with and without the UMI tags on water samples from all the tanks to determine the optimal cluster size cut-off. Since false haplotypes result from sequencing errors, for the most abundant haplotypes, the changes in sensitivity and specificity depend on the cut-off for low-frequency haplotypes. The sequences and frequencies of haplotypes are shown in Supplementary Information Table S2, and we confirmed that the true haplotype sequences with the UMI tags were identical to those without the UMI tags. The data from each tank was normalized by the maximum haplotype cluster size and the sensitivities of haplotype detection are shown in Fig. 3A. Here, the sensitivity is the percentage of correct haplotypes among the total haplotypes observed. The sensitivity in HaCeD-Seq was not different between those with and without the UMI tags, although it tended to be slightly higher with the UMI tags. This indicates that the UMI tags remove the PCR bias caused by difference in the concentrations of different haplotypes that had been amplified by PCR.

**Table 3:** The number of D-loop haplotypes determined based on UMI-assisted HaCeD-Seq.

Group	Individuals	Unique haplotypes	Haplotypes / Individuals (%)	Duplicated haplotypes (a/a)	Duplicated haplotypes (a/b)	Duplicated haplotypes (b/b)	Triplicated haplotypes (b/b)	Haplotype diversity
Previously present (a)	38	36*	95	2	4	–	–	0.991
Newly added (b)	70	64**	91	–	4	4	1	0.990
Total	108	96***	89	2	4	4	1	0.993

a/a, a/b and b/b indicate duplicated or triplicated haplotypes in the aquarium tank, the aquarium tank/vehicle tanks, and vehicle tanks, respectively.

*Determined by NGS with the UMI tags from eDNA samples.

**Determined by Sanger sequencing from tissue samples.

***Total number of haplotypes including previously present and newly added.

**Figure 4:**
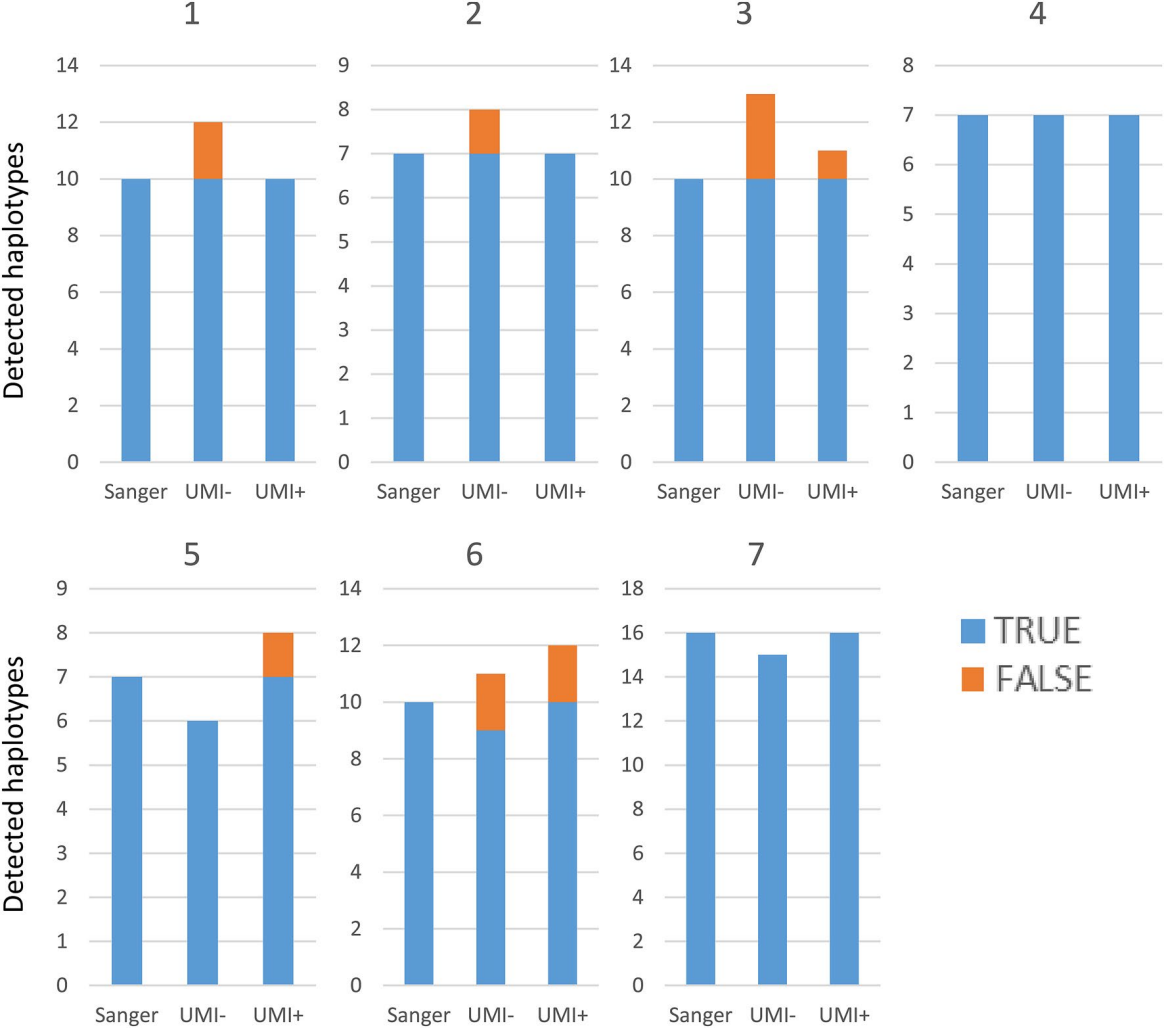
Number of detected haplotypes at optimal threshold. Number of haplotypes detected using a threshold of 0.2% with the UMI tags and 1.5% without the UMI tags; TRUE is the number of correct haplotypes detected; FALSE is the number of false haplotypes detected. Tank 1, 10 haplotypes for 11 individuals; Tank 2, 7 haplotypes for 7 individuals; Tank 3, 10 haplotypes for 11 individuals; Tank 4, 7 haplotypes for 7 individuals; Tank 5, 7 haplotypes for 7 individuals; Tank 6, 10 haplotypes for 10 individuals; Tank 7: 16 haplotypes for 17 individuals.

The specificity results are shown in Fig. 3B. Here, the specificity is the percentage of correct haplotypes compared to all the detected haplotypes. The specificity varied markedly depending on whether the UMI tags were used. We determined the minimum percentage that would result in a specificity of at least 85% as the cut-off. Without the UMI tags, the specificity decreased to below the detection threshold of 2%. By contrast, with the UMI tags, the specificity remained almost unchanged until reaching 0.2%. We chose a threshold of 0.2% with the UMI tags and 1.5% without the UMI tags. The HaCeD-Seq results of the transport vehicle water tank samples using these thresholds are shown in Fig. 4. All of the true haplotypes (determined by Sanger sequencing) were detected both with and without the UMI tags, with exception of tank 6 where only 9/10 true haplotypes were detected without the UMI tags. Across all tanks, the absence of the UMI tags generated 8 false haplotype detections, compared to 4 with the UMI tags.

### Estimation of tuna abundance in the aquarium tank

We next conducted the experiment in the aquarium and compared the results obtained from HaCeD-Seq with and without the UMI tags. A total of 38 tuna were already present in the aquarium tank, and then the 70 tuna were newly added. It was not feasible to determine the haplotypes of the existing 38 tuna by Sanger sequencing because it was difficult to collect tissue samples from live specimens in the large aquarium tank. Therefore, the water in the aquarium containing the 38 tuna was sampled, and 36 haplotypes were successfully obtained from an analysis with the UMI tags (Table 3 and “before” sequences in Supplementary Information Table S1). Among these 36 haplotypes, four were identical to those of fish in the transport vehicle tanks. Thus, together with the additional 70 tuna with 64 haplotypes, the total number of unique haplotypes of the total 108 tuna in the aquarium was 96 (Table 3, Supplementary Information Table S1).

**Figure 5:**
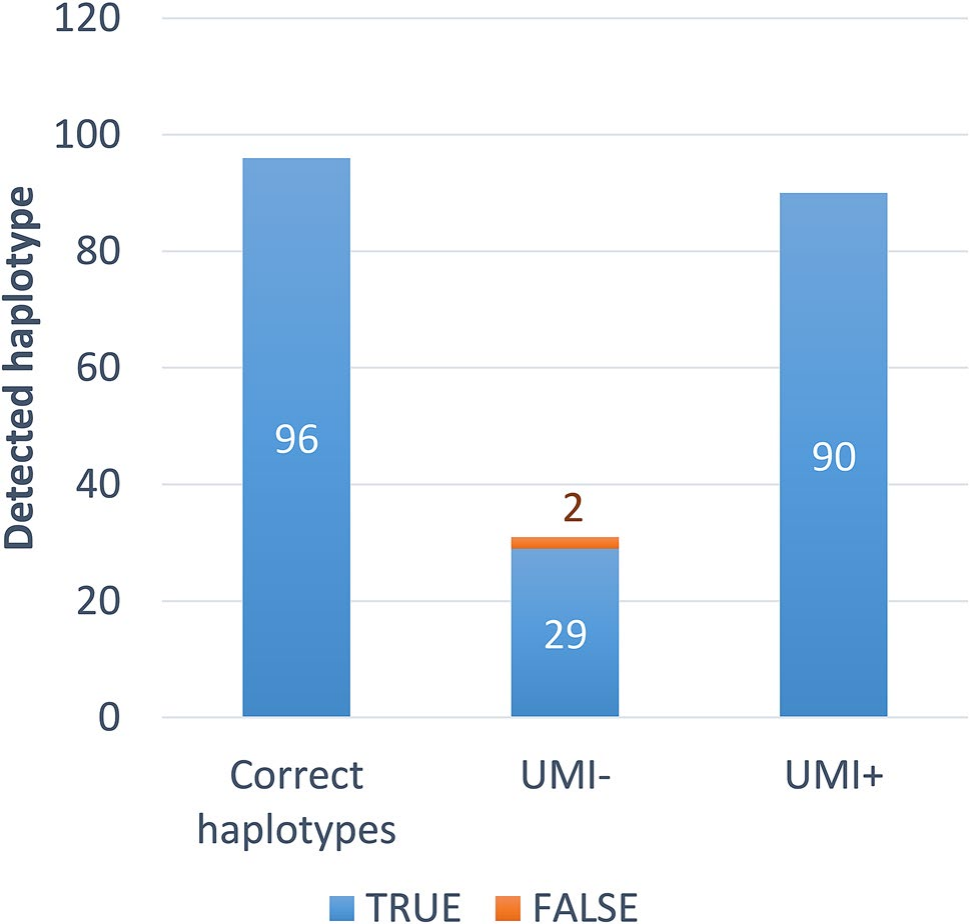
HaCeD-Seq analysis results for the aquarium tank water. The correct number of haplotypes is 96 but only 90 haplotypes were detected when using the UMI tags (UMI+). Only 29 haplotypes and two false haplotypes were detected without the UMI tags (UMI−).

The HaCeD-Seq analysis of water samples from the aquarium tank detected 90 out of the 96 haplotypes (94%) when the UMI tags were used (Fig. 5). However, HaCeD-Seq analysis without the UMI tags detected only 29 haplotypes (30%), with two false haplotypes. These results show that when a large number of haplotypes need to be detected (i.e., greater than 100), the UMI tags are essential to obtain the correct number of haplotypes.

## Discussion

In this paper, we report the first application of a random sequence (unique molecular identifier: UMI) to eDNA analysis. In this method, the D-loop is PCR-amplified as the target region by adding the UMI tags to the end of this region with eDNA as a template. The D-loop and UMI sequences are then determined by next-generation sequencing, and identical DNA sequences with the same UMI sequence are counted as one unique sequence. The reads with different UMI tags are considered to be derived from different DNA copies; by counting the number of UMI tags, an accurate number of DNA copies can be obtained. Furthermore, PCR and sequencing errors can be avoided by integrating sequences with the same UMI tag, and more accurate D-loop sequences can be obtained referring to the data previously reported^14–16,27,28^.

The accuracy of DNA polymerase in DNA synthesis is approximately 99.995%, including that of high-fidelity DNA polymerase^29^. However, as sequencing accuracies have increased over the years, PacBio's HiFi reads exceed 99.99% accuracy, so the effect of PCR errors during library construction has become non-negligible. PCR errors can be also avoided by integrating the results of multiple PCR runs operated in separate tubes. However, because there are cases in which PCR errors might occur at particular sites with similar frequencies even in separate reaction tubes with the same template DNA^27,28^, the removal of PCR errors by software has limitations. For greater accuracy, not only software processing, but also physical processing must be used in combination. We have successfully applied UMI tags to reduce the noise from PCR and next-generation sequencing errors by an order of magnitude when counting haplotypes; these UMI tags were very practical for eliminating PCR errors^14–16^.

By using HaCeD-Seq combined with the UMI tags to estimate the number of overlapping haplotypes in the Pacific bluefin tuna rearing in the aquarium tank at Tokyo Sea Life Park, we were able to detect 90 out of 96 haplotypes (94%) with no false haplotype. These results suggest that almost all the haplotypes present in the aquarium tank could be detected. On the other hand, 4 false haplotypes were detected among 71 haplotypes in 7 vehicle tanks with the UMI tags, suggesting the effect of different DNA concentrations in different sizes of tanks even on the number of false haplotypes for the UMI-assisted method. Meanwhile, only 29 out of 96 haplotypes (30%) could be detected with two false haplotypes without the UMI tags. Nevertheless, we were unable to detect 6 out of the 96 haplotypes (6%). Because 6 individuals died during the first month after addition to the aquarium tank, the six missing haplotypes may have resulted from these deaths. Therefore, it is likely that our haplotype detection rate was greater than 94%. The improved detection sensitivity when using the UMI tags is an important achievement to address the challenge of determining fish abundances from open seawater samples because the variability in concentration among haplotypes is expected to be greater than tank experiments. HaCeD-Seq with the UMI tags is likely to be adopted in the studies of the species living in open seawater with high haplotype diversity.

**Figure 6:**
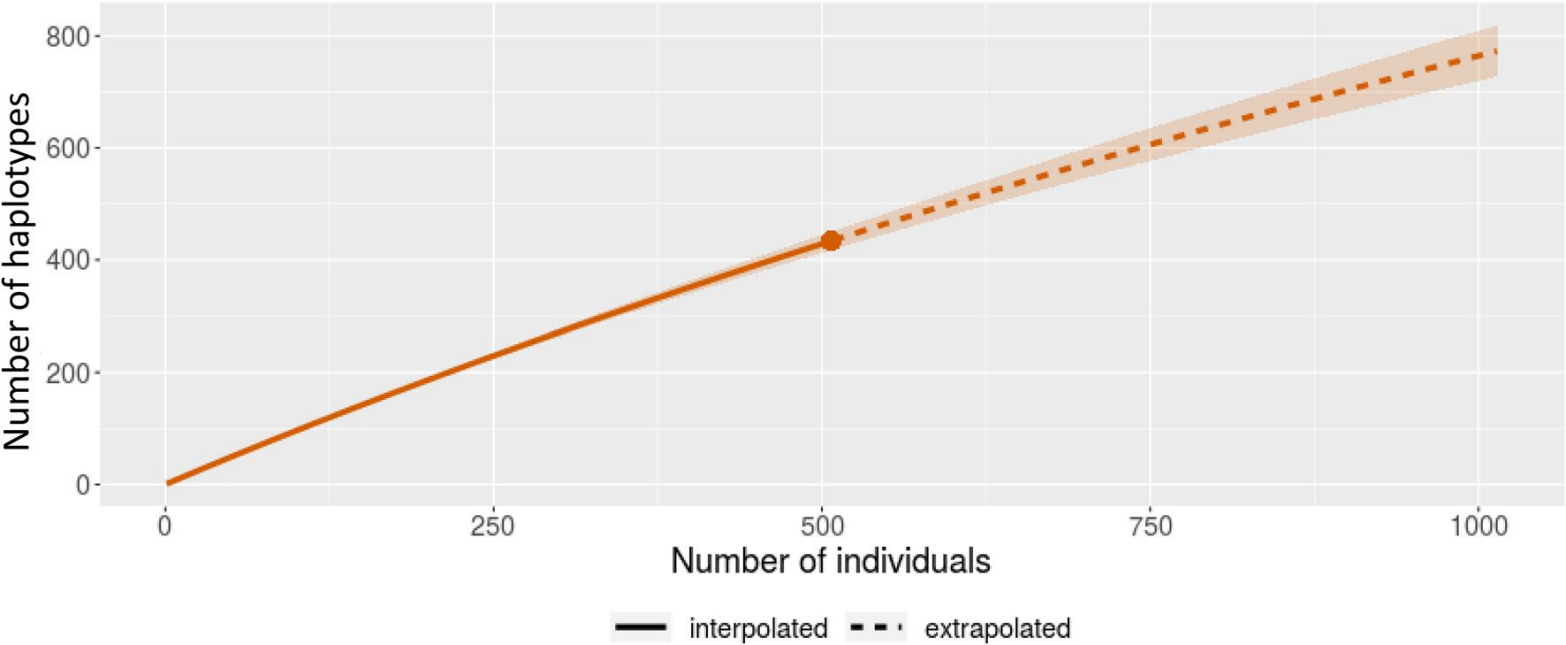
Relationship between the number of individuals and the number of unique haplotypes. Estimation using iNEXT from data on 507 Pacific bluefin tuna (DDBJ accession number: AB535754-AB536538).

Although the haplotype diversity of tuna is high^17,18^, only 96 haplotypes were detected in samples from the aquarium which housed 108 individuals. This discrepancy shows that there are differences between the number of haplotypes and the number of individuals. To estimate the abundance more accurately from the number of detected haplotypes, it is necessary to account for overlap in haplotypes among individuals and thus to make estimates based on a population genetic model as well as the effect of different DNA concentrations in open seawater. The iNEXT tool is a method that can produce such an estimate, and it is possible to estimate the error range of the resulting population numbers from the detected haplotype diversity^30^. In the case of Pacific bluefin tuna, the error range was estimated using the D-loop sequence of 507 bluefin tuna in a public database (Fig. 6). For a haplotype number of 96, the estimated number of individuals ranged from 98 to 101. These results will be an important guideline for setting research policies for field studies.

HaCeD-Seq makes it possible to estimate the number of individual fish in a tank from the eDNA contained in a water sample. Some fish hide behind rocks inside the aquarium, and even fish keepers are often unsure of the number of individuals in a given tank. Most fish in aquariums are wild-caught individuals, and the haplotype diversity is expected to be high. Therefore, the method tested in this study can also be applied to monitoring the fish abundance in aquarium tanks as well.

The extent of exploitation of commercially important fish has been increasing due to increasing global demand. In particular, the global depletion of top predatory fish species such as the Pacific bluefin tuna is a serious concern in the context of sustainable fisheries management^31,32^. The estimation of the abundance and biomass (i.e., stock assessment) of fishery resources has heavily depended on fishery information, such as catch data from global stock assessment systems. A variety of resource estimation models have been devised to improve the accuracy of conventional resource estimation, which relies on catch data. The close-kin method, which uses kinship information, has been newly established for Pacific bluefin tuna and southern bluefin tuna^33,34^. This method can estimate the absolute abundance of parental fish based on the number of parent–offspring pairs determined through DNA marker-based parentage analysis. However, it is critical to collect specimens without bias in terms of distribution, migration, spawning time and location. Samples for the close-kin method are collected by fishery-independent sampling surveys or from marketplaces. Therefore, the reliability of this method depends on the accuracy of catch information. By contrast, the method proposed in the current study does not require fish sampling because it uses the eDNA contained in environmental water samples to estimate the abundance of a target species. In addition, as the conventional method of stock assessment relies on fisheries, many individuals are to be missed. High haplotype diversity has already been reported for some commercially important small pelagic fish such as jack mackerel (*Trachurus japonicus*)^35^, chub mackerel (*Scomber japonicus*)^36^, and spotted mackerel (*Scomber australasicus*)^37^ for which a total allowable catch (TAC) has been set in the fishery management system of Japan^38^. The haplotype diversities of jack mackerel, chub mackerel and spotted mackerel have been reported to be 0.964–1.000, 0.505–0.967 and 0.996, respectively. Hence, the proposed method could be applicable for stock assessments of these major target species with high haplotype diversity in fisheries. It is interesting to investigate how high haplotype diversity is needed to apply this approach. A combination of the proposed method and the conventional methods is expected to improve the accuracy of estimating the abundance and biomass of aquatic organisms. Furthermore, this method would also potentially be applicable to any commercial or non-commercial species if the estimation process is first established based on haplotype diversity. From such a viewpoint, this method is anticipated to provide a possible venue for multispecies approach under the conceptual framework of ecosystem-based fishery management^39^, which is a new direction for the management of fisheries that places priority on the ecosystem rather than on a specific target species.

There are many studies showing the correlation between the abundance of eDNA and the biomass^5–7^. However, abundance estimation in the natural environment has been difficult, because the concentrations of eDNA are variable by variations in size and age-class distributions even in the same species^8^ as well as alteration in environmental factors such as water temperature and flow^9,40,41^. This has been an open question linking eDNA to species abundance. Yamamoto et al.^42^ used eDNA with quantitative PCR in combination with echo sounder technology to estimate the distribution and biomass of jack mackerel. Our method allows the fish abundance and biomass to be estimated from environmental water samples without any further supplementary method. Such methodological independence is a strong advantage of our approach, which establishes a simple and accurate estimation of fish abundance. Although different numbers of the sequences were distributed across different haplotypes with the UMI tagged data, we can detect almost 100% of the haplotypes. This data would allow us to see how variable eDNA production rates amongst individuals of the same species, which we think would be a very useful addition to our knowledge.

One of the most suitable applications of the present method is to estimate the number of individuals of endangered species. Endangered species are rapidly declining in abundance and biomass, making it more difficult to sample individuals in the field. A direct abundance estimation based on our method using only environmental water samples could be a desirable way to assess the population status of endangered species without disturbing the ecosystem through field sampling. Our proposed method provides a route for the simultaneous pursuit of sustainable management, ecosystem conservation, and necessary assessment.

## Materials and methods

### Specimens

Pacific bluefin tuna were caught in the Pacific Ocean off Kashiwa Island, Otsuki, Kochi Prefecture; off Kannoura, Toyo, Kochi Prefecture; and off Nachikatsuura, Wakayama Prefecture. All specimens were temporarily kept in cages off Kashiwa Island and then transported by a live fish boat to Misaki, Kanagawa Prefecture. From this location, four live fish transport vehicles with a combined total of seven tanks were used to transport 70 tuna to the Tokyo Sea Life Park on June 12, 2016. Each transport tank (weighing 7–17.1 tons) contained 7–17 tuna (7, 7, 7, 10, 11, 11 and 17 tuna) (Fig. 1). While these transport tanks had no circulation system, aeration with pure oxygen and air were provided. No fish deaths occurred during transportation from Misaki to the park or during transfer from the vehicle tanks to the aquarium. A 10 L sample of environmental water was collected from each tank after transportation. A total of 38 Pacific bluefin tuna were currently housed in a 2,200-ton aquarium tank at the park, and a 10 L sample of the surface water from this tank was collected on June 8, 2016, before adding the 70 new Pacific bluefin tuna. The water temperature of the aquarium tank was 23 °C, and the circulation volume was 2,200,000 L/h. About 60% of the water in the aquarium tank was replaced per month. The water of fresh natural seawater was taken off Hachijojima in Izu Islands and transported to the aquarium. Aeration with pure oxygen and air were provided, where dissolved oxygen concentration was maintained at 6.3–6.7 mg/L. Pieces of dorsal fin tissues of the tuna were dissected with small forceps from the 70 individuals to determine the D-loop sequences by Sanger sequencing, and pit tags were embedded for individual identification. One month after the addition of the 70 tuna, 10 L of surface water was again collected from the aquarium tank on July 6, 2016. The dates of the individuals that died in the aquarium tank between June 8 and July 6, 2016, are as follows. Two individuals previously present in the aquarium tank died on June 13 and June 27, 2016. Four individuals newly added died in the aquarium tank each on June 13, June 14, June 14, and June 16, 2016. All animal handling methods and experimental protocols were carried out in accordance with the Guidelines for Animal Experimentation at Fisheries Research Agency. This study was approved by the subcommittee on institutional animal care and use of Graduate School of Agricultural and Life Sciences, The University of Tokyo (permission # P14-952).

### DNA extraction and sanger sequencing for reference individuals

For each of the aforementioned 70 individuals (reference individuals), the total DNA to be used for PCR amplification of the whole D-loop was extracted from the excised fin tissue using the DNeasy Blood & Tissue Kit (Qiagen). The DNA concentration was quantified with a Qubit dsDNA BR Assay Kit (Invitrogen, Thermo Fisher Scientific).

The PCR primer design in Primer3^43^ was based on a complete mitogenome sequence of Pacific bluefin tuna obtained from the NCBI nucleotide database (accession: AB185022). In the mitogenome sequence, the forward primer (ToCR3_F: 5′-CAGGCTGAGCTGAGAACAAA-3′) spans from the 17th to 36th upstream site of the 3′-end of the cytochrome *b* gene, whereas the reverse primer (ToCR1_R: 5′- GGGCCCATCTTAACATCTTCA-3′) is positioned within the tRNA^Phe^ gene. Hence, the primer set is capable of amplifying the whole tRNA^Thr^, tRNA^Pro^, and D-loop (expected amplicon size of 1,092 bases, including 865 in the D-loop in the mitogenome sequence).

PrimeSTAR GXL polymerase (Takara Bio) was used in the PCR reaction. The PCR reaction solution (25 μl total volume) contained 5.0 µL of 5× buffer provided by the supplier, 2.0 µL of dNTP mix (2.5 mM each), 1.0 µL each of the ToCR3_F and ToCR1_R primers (10 µM), 0.5 µL of polymerase (1.25 units/µL), 13.5 µL of ddH_2_O, and 2.0 µL of DNA solution (10 ng/μl). Thermal cycling in a ProFlex PCR System (Applied Biosystems, Thermo Fisher Scientific) consisted of 35 cycles of 98 °C for 10 s, 60 °C for 15 s, and 68 °C for 1 min. The amplified products were enzymatically purified with exonuclease I (New England Biolabs) and thermo-sensitive alkaline phosphatase (Promega), as previously described^44^. The PCR primers were used to sequence the amplicons from both directions using a BigDye Terminator v3.1 Cycle Sequencing Kit in combination with a 3730xl DNA Analyzer (Applied Biosystems, Thermo Fisher Scientific). The resulting sequences were aligned and edited in DNASIS Pro version 2.02 (Hitachi Software Engineering). Multiple sequence alignment was conducted using the ClustalW algorithm implemented in MEGA6^45^. After multiple sequence alignment, a stretch of 365 bases of the D-loop sequence determined for each individual served as a reference sequence in downstream eDNA sequencing.

### Extraction of environmental DNA from water

A 10 L sample of collected environmental water was passed through a 47 mm GF/D (2.7 μm pore size) glass-fibre filter (Whatman). After filtering the samples, the filters were fixed by adding 15 mL of ethanol^46^. The filter was divided into two equal parts with scissors before DNA extraction; one of the two pieces was used for DNA extraction in order to properly fit the extraction kit. DNA extraction from the filter was performed using a DNeasy Blood & Tissue Kit^47^. The extracted eDNA was eluted in 400 µL (twice with 200 µL) of buffer AE supplied with the kit. The extracted eDNA was quantified using a Qubit 2.0 Fluorometer (Thermo Fisher Scientific) and subjected to PCR under the same conditions as used for Sanger sequencing to confirm PCR amplification.

### UMI-tagged PCR and MiSeq sequencing

To construct the UMI-tagged libraries, another primer set (degenerate primers) was designed within the D-loop region: To2nd_CRF2 (5′-CTAGTACYYAACCATTCATA-3′) and To2nd_CRR1 (TGACCCYCTAGARAGAACG-3′). The extracted eDNA was adjusted for UMI-tagged libraries according to the following procedures. The construction of the UMI-tagged libraries was performed in a three-step PCR protocol (Supplementary Information Fig. S1).

For the first PCR, 10 ng of DNA was used as a template, and To2nd_CRF2_CS_UMI13: 5′-CTCTTCCGATCTGTCNNNNNNNNNNNNNCTAGTACYYAACCATTCATA-3′ and To2nd_CRR1_withCS: 5′-CTCTTCCGATCTCAGTGACCCYCTAGARAGAACG-3′ were used as primers. The composition of the PCR reaction medium (25 µL total) was 0.5 µL of PrimeSTAR GXL DNA Polymerase, 5.0 µL of 5× Prime STAR GXL Buffer, 2.0 µL of dNTP mixture (2.5 mM each), 1.0 µL of primer To2nd_CRF2_CS_UMI13 (10 µM), 1.0 µL of primer To2nd_CRR1_withCS (10 µM), 10 ng of sample, and adjusted to 15.5 µL with distilled water. Four cycles of 10 s at 98 °C, 30 s at 55 °C and 1 min at 68 °C were carried out in a thermal cycler, and the reaction solution was then stored at 4 °C. Next, 0.1 µL of exonuclease I (Takara Bio), 3 µL of 10× reaction buffer (included with the exonuclease), and 1.9 µL of nuclease-free water were added for a total of 30 µL, and the reaction was carried out at 37 °C for 30 min to remove the primers. The exonuclease was then inactivated at 80 °C for 20 min, and 10 µL of distilled water was added to bring the total volume to 40 µL. The post-reaction mixture was treated with AMPure XP beads (Beckman Coulter), and PCR amplicons were eluted in 17 µL of 10 mM Tris–HCl buffer (pH 8.0), 15.5 µL of which was collected and used as sample for the second PCR.

The second PCR was performed with the primers CS1-24 bp-F: 5′-TACACGACGCTCTTCCGATCTGTC-3′, CS2-24 bp-R: 5′-AGACGTGTGCTCTTCCGATCTCAG-3′ at a final concentration of 400 nM. The PCR reaction solution (25 µL total volume) was composed of 0.5 µL of PrimeSTAR GXL DNA Polymerase, 5.0 µL of 5× PrimeSTAR GXL Buffer, 2.0 µL of dNTP mixture (2.5 mM each), 1.0 µL of primer CS1-24 bp-F (10 µM), 1.0 µL of primer CS2-24 bp-R (10 µM) and 15.5 µL of sample. After adjusting the reaction solution, five cycles of 10 s at 98 °C, 15 s at 50 °C, and 30 s at 68 °C were performed in a thermal cycler, followed by 10 cycles of 10 s at 98 °C, 15 s at 60 °C, and 30 s at 68 °C. The samples were then stored at 4 °C. Subsequently, the samples were purified using AMPure XP beads as in the first PCR. The third PCR was performed with the primers PE1-CS1-ID50X: 5′-AATGATACGGCGACCACCGAGATCTACACXXXXXXXXACACTCTTTCCCTACACGACGCTCTTCCGATCTGTC-3′, PE2-CS2-ID70X: 5′-CAAGCAGAAGACGGCATACGAGATXXXXXXXXGTGACTGGAGTTCAGACGTGTGCTCTTCCGATCTCAG-3′ (XXXXXXXX: Illumina multiplex tags, Illumina) at a final concentration of 400 nM. The PCR reaction solution (25 µL total volume) was composed of 0.5 µL of PrimeSTAR GXL DNA Polymerase, 5.0 µL of 5× PrimeSTAR GXL Buffer, 2.0 µL of dNTP mixture (2.5 mM each), 1.0 µL of primer PE1-CS1-ID50X (10 µM), 1.0 µL of primer PE2-CS2-ID70X (10 µM) and 15.5 µL of sample. After adjusting the reaction solution, five cycles of 10 s at 98 °C, 15 s at 60 °C, and 30 s at 68 °C were performed in a thermal cycler.

Library construction without the UMI tag was performed with the primers To2nd_CRF2_withCS: CTCTTCCGATCTGTCCTAGTACYYAACCATTCATA and To2nd_CRR1_withCS: CTCTTCCGATCTCAGTGACCCYCTAGARAGAACG as in the first PCR. The subsequent procedures were performed under the same conditions as in the UMI library preparation. The constructed libraries were paired-end sequenced at 600 bp on a MiSeq next-generation sequencer (Illumina).

### HaCeD-Seq analytical pipeline

#### Without UMI tags

After joining the paired-end reads with FLASh ver 1.2.11^48^ using the option “-M 300”, 20 nucleotides from the 5′-end and 22 nucleotides from the 3′-end were deleted as the adapter and primers region, and the reads that contained one or more < Q30 bases were pruned using a self-made script. The variety and abundance of each haplotype was calculated by clustering the remaining reads at 100% agreement.

#### With UMI tags

After joining the paired-end reads with FLASh ver. 1.2.11 using the option “-M 300”, MAGERI ver. 1.1.1 was run with the following command:

java -Xmx32G -jar mageri.jar -M3 nnnNNNNNNNNNNNNN -R1 LibX.extendedFrags.fastq -references ref.fa -sample-name LibX.

Next, 20 nucleotides from the 5′-end and 22 nucleotides from the 3′-end were deleted as the adapter and primers regions of the obtained consensus sequence, and the number of 100% matching consensus sequences was calculated for each haplotype.

## Supplementary Information

Supplementary Information 1.

Supplementary Table S1.

Supplementary Table S2.

## Data Availability

All MiSeq sequencing data were registered in the DNA Data Base of Japan (DDBJ) under accession number DRA010682.

## Abbreviations

HaCeD: Haplotype count from eDNA
