# Supplementary material for: Estimation of tuna population by the improved analytical pipeline of unique molecular identifier-assisted HaCeD-Seq (haplotype count from eDNA)
Source: Sci Rep. 2021 Apr 12;11:7031. doi: 10.1038/s41598-021-86190-6 (PMC8041778; doi:10.1038/s41598-021-86190-6)
Supplement: Supplementary file 1 — Supplementary Information 1. [file 41598_2021_86190_MOESM1_ESM.docx]

**Estimation of tuna population by the improved analytical pipeline of unique molecular identifier-assisted HaCeD-Seq (haplotype count from eDNA)**

**Kazutoshi Yoshitake^1^, Atushi Fujiwara^2^, Aiko Matsuura^3^, Masashi Sekino^3^, Motoshige Yasuike^3^, Yoji Nakamura^3^, Reichiro Nakamichi^3^, Masaaki Kodama^4^, Yumiko Takahama^4^, Akinori Takasuka^1^, Shuichi Asakawa^1^, Kazuomi Nishikiori^4^, Takanori Kobayashi^5,6^, Shugo Watabe^6,*^**

^1^ Department of Aquatic Bioscience, Graduate School of Agricultural and Life Sciences, The University of Tokyo, 1-1-1 Yayoi, Bunkyo-ku, Tokyo 113-8657, Japan. ^2^ Fisheries Technology Institute, Japan Fisheries Research and Education Agency, 422-1 Nakatsuhamaura, Minami-ise, Mie, 516-0193, Japan ^3^ Fisheries Resources Institute, Japan Fisheries Research and Education Agency, 2-12-4 Fuku-ura, Kanazawa, Yokohama, Kanagawa 236-8648, Japan ^4^ Tokyo Sea Life Park, 6-2-3 Rinkai-cho, Edogawa-ku, Tokyo 134-8587, Japan ^5^ Japan Fisheries Research and Education Agency, 2-3-3 Minato Mirai, Nishi-ku, Yokohama, Kanagawa 220-6115, Japan ^6^ School of Marine Biosciences, Kitasato University, 1-15-1 Kitasato, Minami-ku, Sagamihara, Kanagawa 252-0373, Japan. *e-mail: [swatabe@kitasato-u.ac.jp](mailto:swatabe@kitasato-u.ac.jp)

**Supplementary information**

**Supplementary Information Table S1**. Supplementary Informatin Table S1. Haplotypes based on D-loop sequences of Pacific bluefin tuna examined in the present study. "before" indicates haplotypes of 30 tuna which existed in the aquarium tank before adding 70 juveniles and "tank", those of 70 juveniles newly added from vehicle tanks.

**Supplementary Information Table S2**. Cluster sizes of haplotypes. No.1-7 indicate vehicle tanks. "before"and "after" indicate tuna samples which existed in the aquarium. tank before and after adding 70 juveniles, respectively.

**Supplementary Information Figure S1.** The construction of the UMI-tagged libraries in a three-step PCR protocol.

Supplementary Information Figure S1.
